# Predicting susceptibility to COVID-19 infection in patients on maintenance hemodialysis by cross-coupling soluble ACE2 concentration with lymphocyte count: an algorithmic approach

**DOI:** 10.3389/fmed.2024.1444719

**Published:** 2024-10-30

**Authors:** Shuang Yuan, FuLei Meng, Shuai Zhou, XiaoYing Liu, XiaoMing Liu, LiHong Zhang, Tao Wang

**Affiliations:** ^1^Graduate School of Hebei Medical University, Shijiazhuang, China; ^2^Department of Nephrology, The First Hospital of Hebei Medical University, Shijiazhuang, China

**Keywords:** COVID-19, maintenance hemodialysis, ACE2, TMPRSS2, lymphocyte count

## Abstract

**Introduction:**

Patients on maintenance hemodialysis (MHD) were more vulnerable to and had a higher mortality during the COVID-19 pandemic. As angiotensin converting enzyme 2 (ACE2) and transmembrane protease serine S1 member 2 (TMPRSS2) played crucial roles in viral entry into the human host cells, we therefore investigated in the MHD patients whether their plasma levels were associated with susceptibility to the COVID-19.

**Methods:**

Blood samples were collected from the patients in our then COVID-19 free center immediately upon lifting of the stringent quarantine measures in early December of 2022 and infection situation was observed within the following 2 weeks. Plasma levels of the soluble ACE2 (sACE2), ACE (sACE) and TMPRSS2 (sTMPRSS2) were measured with ELISA method. Data were stepwisely tested for independent effect, relevant role and synergistic action on the susceptibility by multiple logistic regression, receiver operating characteristic curve and multiple dimensionality reduction (MDR) method, respectively.

**Results:**

Among the 174 eligible patients, 95 (54.6%) turned COVID-19 positive with a male to female ratio of 1.57 during the observation period. Comparing with the uninfected, the infected had significantly higher sACE2 and lower sTMPRSS2 levels upon comparable sACE concentration. Besides the sACE2, factors associated with susceptibility were vintage and individual session time of the hemodialysis, smoking and comorbidity of hepatitis, whereas lymphocyte counts showed a tendency (*p* = 0.052). Patients simultaneously manifesting higher sACE2 level and lower lymphocyte counts had an increased infection risk as confirmed by the MDR method.

**Conclusion:**

By sorting out the susceptible ones expeditiously, this algorithmic approach may help the otherwise vulnerable MHD patients weather over future wave of COVID-19 variants or outbreak of other viral diseases.

## Introduction

Coronavirus disease 2019 (COVID-19), caused by severe acute respiratory syndrome coronavirus 2 (SARS-CoV-2), has delivered an almost devastating blow to the world, leaving millions of death in its wake. It is now clear that entry of the virus into host cells depends on two enzymes: angiotensin converting enzyme 2 (ACE2) and transmembrane protease serine S1 member 2 (TMPRSS2) (1). Specifically, the viral spike protein binds to the ACE2 and forms the virus-ACE2 complex. The TMPRSS2, which is expressed by human respiratory, gastrointestinal and urogenital epithelium, then cleaves the spike protein subunits 1 and 2, thus enabling the direct fusion of S2 subunit with the targeted cell membrane and viral entry (2).

Study using primary human airway epithelial cells from German subjects has congruently confirmed the role of ACE2 as receptor and TMPRSS2 as facilitator for cell entry of the SARS-COV-2 (3). Conceivably, natural mutation in and artificial action on the ACE2 may confer resistance to (4) and therapeutic option of the COVID-19 (5), respectively. In this regard, graphene-derived products were suggested to be capable of preventing the COVID-19 infection (6). Detailed discussion of these issues was further available elsewhere (7). Similarly, TMPRSS2 inhibition was suggested as a prophylactic and therapeutic option against the COVID-19 (8). Moreover, lymphocyte dysfunction was also associated with susceptibility to the virus infection (9).

Patients on maintenance hemodialysis (MHD) was a high risk population for COVID-19 and the mortality rate varied between 20 and 35% which was more than twice higher than that in the general population (10). As such, one of the major lessons was the inability to effectively predict who had the higher risk of infection to enable proper risk stratification and early intervention. Consistently, the UK Health Security Agency released a summative study this May of focusing on non-pharmaceutical interventions to reduce COVID-19 transmission (11). This report essentially highlighted the fact that the pandemic but not evolution of the SARS-CoV-2 was over. By nature, the rates of nucleotide substitution of SARS-COV-2 are fast. This higher error rate and the consequent rapidly evolving virus populations, which could lead to the accumulation of amino acid mutations, might affect the transmissibility of the virus, its cell tropism and pathogenicity (12). Indeed, the JN.1-derived KP.2 variant has sprouted out (7) most recently along with the resurgence of highly pathogenic avian influenza A (H5N1) virus (13). Thus, finding a way to identify those MHD patients susceptible to virus infections is of great importance, even today.

In the present study, we therefore sought to evaluate levels of both circulating soluble ACE2 (sACE2) and TMPRSS2 (sTMPRSS2) in MHD patients and examine how they could be used with the lymphocyte count in risk prediction. Similar to our previous work of predicting model for pulmonary infection in patients with membranous nephropathy taking the cyclosporin regime (14), an algorithmic approach for risk assessment may arguably increase the preparedness for possible wave of the COVID-19 variants or other global viral disease outbreak.

## Methods

### Study design

The study was conducted at the hemodialysis center in the First Hospital of Hebei Medical University and the subjects were those on in-center MHD. Generally, the subjects were at least 18-year-old when initiated the MHD and had a dialysis vintage for more than 6 months, without history of prior renal transplantation, free of the virus infection, absent from malignancy and taking no immunosuppressant, as previously described (15). The dialysis prescription was made according to the KDIGO guidelines, with the only exception in duration of each dialysis session. They received either 3 or 4 h in each session as dictated by the requirement of quarantine and availability of dialysis staffs. Otherwise, anemia, hypertension and hyperphosphatemia were managed by standard protocols, whereas low molecular weight heparin was used for anticoagulation. Medical history, medications and lifestyle factors were recorded by the attending dialysis staffs.

### Study timing and oversight

The study was launched immediately upon changes in the quarantine measures in early December of 2022 when all the subjects were negative of COVID-19 and infection status was observed within the following 2 weeks. The diagnosis of infection was made once SARS-CoV-2 RNA was confirmed by reverse-transcriptase polymerase chain reaction (RT-PCR) assay and the remaining COVID-19 negative patients served as control cohort. Prior to the start, the study was approved by our institutional review board and all patients gave written informed consent.

### Laboratory tests

Venous blood was collected before the second hemodialysis session of the week after overnight fasting. Data of blood routine was acquired using Beckman Coulter cellular analysis system (Unicel DxH800, CA, United States). Plasma parameters were measured by using Beckman Coulter AU5800 automatic biochemical analyzer. Ferritin and intact parathyroid hormone (iPTH) were determined by Beckman Coulter automatic chemiluminescence immunoassay analyzer (UniCel DxI800). Kt/V of the hemodialysis was derived from the well-established KDOQI equation.

### Measurements of the plasma level of sACE, sACE2 and sTMPRSS2

Circulating levels of ACE2, ACE and TMPRSS2 from plasma samples were quantified using the Abcam Human SimpleStep ELISA^®^ Kit (Cambridge, MA, United States) (16), following the manufacturer’s instructions. Major technical parameters for the ACE2 kit (ab235649) are: range of detection 3.98–255 ng/mL, sensitivity 1,052 pg./mL, intra-assay CV 2.3% and inter-assay CV 3.2%. In the same order, corresponding values for the ACE kit (ab263889) are 0.625–40 ng/mL, 0.15 ng/mL, 3.4 and 3.0%. For the TMPRSS2 kit (ab283552), they were 15.625–1,000 pg./mL, 4.266 pg./mL, 4.9 and 5.8%. The OD at 450 nm was determined on a multiskan MK3 reader (Thermo Scientific, CA, United States). Each measurement was performed in duplicate and average value was used.

### Statistical analysis

Statistical analyses were performed using SPSS version 19.0 (SPSS, Chicago, IL, United States). All data used in the analysis were normally distributed as the significantly skewed ones were log-transformed. Student’s *t-*test and the chi-square test were used for comparing continuous and categorical variables between groups, respectively. Multiple logistic regression analysis was then used to examine the independent effect of sACE/sACE2/sTMPRSS2, if any, on the susceptibility with adjustment of potential confounding factors. The selection of confounding factors was previously described in details (17), which mainly depended on clinical relevance and results of preceding *t*-test. The identified risk factors were further evaluated by the receiver operating characteristic (ROC) curve, which generated paired sensitivity/specificity ranking and the optimal one (cutoff value) was selected according to the Youden’s index (18). Finally, interaction between risk factors that may influence the susceptibility was examined by the multifactor dimensionality reduction (MDR) method. Subjects are divided into high-and low-risk groups, using the individual cutoff value of risk factors, and the MDR method is able to detect significant inter-group difference through cross-validation and permutation testing, as we have previously described (19). Two-sided *p <* 0.05 was considered statistically significant.

## Results

After the observation period of 2 weeks, we had 95 patients turned COVID-19 positive and 79 remained negative among our MHD cohorts. The symptoms included fever (60.0%), upper respiratory manifestations (70.5%), digestive tract disturbance (56.8%) and dysgeusia (39.7%). As shown in Table 1, age, gender composition and cause of their ESRD between the two groups were comparable. In terms of dialysis duration, comorbidities, and medication, however, there was significant inter-group difference in hemodialysis vintage of more than 120 months, concurrent HBV/HCV infection, smoking and the use of angiotensin receptor blocker (ARB). Clinical features of the two groups were further listed in Table 2 and the significantly different ones were interdialysis weight gain, lymphocyte count, C-reaction protein, plasma uremic acid, ferritin, dialysis session time and ultrafiltration. Otherwise, there was no difference in the pre-dialysis blood pressure, blood routine, hepatic and renal function, lipid profiles and Kt/V. Moreover, sACE2 but not sACE level was significantly higher in the infection group, whereas the sTMPRSS2 level showed reverse pattern (Table 3).

**Table 1:** General profiles of the infection and non-infection groups.

	With COVID-19	Without COVID-19	*p-*value
No. of patients	95	79	
Age (year)	54.8 ± 14.0	56.4 ± 14.5	0.481
Male	58 (61.1%)	52 (65.8%)	0.516
Cause of ESRD			
Chronic glomerulonephritis	26 (27.4%)	20 (25.3%)	ns
Diabetic nephropathy	28 (29.5%)	23 (29.1%)	ns
Hypertension	22 (8.4%)	17 (21.5%)	ns
ADPKD	6 (2.3%)	6 (7.6%)	ns
Nephrotic syndrome	2 (0.8%)	3 (3.8%)	ns
Obstructive kidney diseases	2 (0.8%)	3 (3.8%)	ns
Unknown	9 (3.4%)	7 (8.9%)	ns
Dialysis vintage (Mo)	60.2 ± 60.4	58.7 ± 47.8	ns
<6 Mo	12 (12.6%)	8 (10.1%)	ns
6–36 Mo	34 (35.8%)	27 (34.2%)	ns
36–120 Mo	36 (37.9%)	37 (46.8%)	ns
**>120 Mo**	13 (13.7%)	7 (8.9%)	**0.001**
Comorbidity			
Hypertension	87 (91.6%)	76 (96.2%)	0.212
Diabetes mellitus	37 (38.9%)	32 (40.5%)	0.834
Coronary heart disease	15 (15.8%)	17 (21.5%)	0.331
Cerebrovascular disease	20 (21.1%)	14 (17.7%)	0.581
COPD	2 (2.1%)	4 (5.1%)	0.287
Digestive tract disease	4 (4.2%)	9 (11.4%)	0.073
**HBV/HCV**	2 (2.1%)	14 (17.7%)	**0.005**
**Smoking**	12 (12.6%)	21 (26.6)	**0.019**
Drinking	13 (13.7%)	14 (17.7)	0.464
Medication			
ACEI	3 (3.2%)	4 (5.1%)	0.532
**ARB**	17 (17.9%)	24 (30.4%)	**0.038**
ARNI	24 (25.3%)	16 (20.3%)	0.415
CCB	69 (72.6%)	64 (81.0%)	0.178
EPO	83 (87.4%)	74 (93.7%)	0.128
Roxadustat	18 (18.9%)	19 (24.1%)	0.408
Active vitamin D3	44 (46.3%)	38 (48.1%)	0.803
Phosphate binder	75 (78.9%)	55 (69.6%)	0.159
Statin	19 (20.0%)	17 (21.5%)	0.801
Anti-platelet agent	11 (11.6%)	10 (12.7%)	0.824
Hypoglycemic drugs	3 (3.2%)	2 (2.5%)	0.807
Insulin	25 (26.3%)	17 (21.5%)	0.466

ESRD, end stage renal diseases; ADPKD, autosomal dominant polycystic kidney disease; Mo, month; COPD, chronic obstructive pulmonary disease; HBV/HCV, hepatitis B and C virus, respectively; ACEI, angiotensin converting enzyme inhibitor; ARB, angiotensin receptor blocker. ARNI, angiotensin receptor neprilysin inhibitor; CCB, calcium channel blocker; EPO, erythropoietin.

**Table 2:** Clinical features of the infection and non-infection groups.

	With COVID-19	Without COVID-19	*P-*value
No. of patients	95	79	
Body mass index (Kg/m^2^)	22.1 ± 3.6	23.6 ± 4.0	0.063
Pre-dialysis SBP (mmHg)	149.6 ± 23.1	152.5 ± 19.7	0.377
Pre-dialysis DBP (mmHg)	84.7 ± 14.1	85.8 ± 12.3	0.637
**Interdialysis weight gain (Kg)**	2.37 ± 1.22	3.02 ± 1.28	**0.001**
White blood cell (×10^9^/L)	5.06 ± 2.58	5.19 ± 1.51	0.692
Neutrophil (×10^9^/L)	3.74 ± 2.48	3.70 ± 1.32	0.911
**Lymphocyte (×10** ^**9**^ **/L)**	0.75 ± 0.39	0.89 ± 0.34	**0.013**
Neutrophil-lymphocyte ratio	5.37 ± 3.68	4.85 ± 2.89	0.117
Hemoglobin (g/L)	111.2 ± 13.5	109.7 ± 14.2	0.464
Platelet (×10^9^/L)	159.9 ± 89.6	156.4 ± 58.9	0.765
**C-reaction protein** ^†^	23.9 ± 40.5	9.75 ± 16.2	**0.004**
Albumin (g/L)	37.1 ± 4.0	37.9 ± 3.1	0.126
GOT (IU/L)	18.3 ± 14.2	15.4 ± 8.9	0.122
GPT (IU/L)	13.0 ± 11.1	14.4 ± 10.0	0.371
Pre-dialysis BUN (mmol/L)	29.0 ± 8.0	28.1 ± 5.1	0.457
Pre-dialysis Scr^†^ (μmol/L)	1172.8 ± 393.0	1048.4 ± 283.5	0.103
Potassium (mmol/L)	5.19 ± 0.75	5.34 ± 0.78	0.201
Calcium (mmol/L)	1.99 ± 0.26	2.06 ± 0.23	**0.053**
Phosphate (mmol/L)	2.23 ± 0.74	2.06 ± 0.60	0.201
**Uremic acid (mmol/L)**	492.3 ± 81.4	454.5 ± 109.9	**0.003**
Cholesterol (mmol/L)	3.90 ± 1.01	4.00 ± 1.21	0.532
Triglyceride^†^ (mmol/L)	2.04 ± 0.90	2.06 ± 1.79	0.922
**Ferritin**^†^ **(ng/mL)**	270.7 ± 256.4	168.1 ± 165.4	**0.003**
Transferrin saturation (%)	24.9 ± 14.3	28.1 ± 15.0	0.146
iPTH^†^ (pg/mL)	395.0 ± 302.2	410.5 ± 295.1	0.734
**Session time (hour)**	3.33 ± 0.36	3.90 ± 0.29	**0.001**
**Ultrafiltration (L)**	2.2 ± 1.0	2.8 ± 1.1	**0.001**
Kt/V^†^	1.26 ± 0.53	1.52 ± 0.82	0.141

Results are given as mean ± SD. Differences between the groups were examined by *χ*^2^ test or unpaired t test when deemed appropriate. *P* < 0.05: significant inter-group difference. ^†^Log-transformed values used in the analysis. SBP and DBP, systolic and diastolic blood pressure, respectively. GOT and GPT, glutamic oxaloacetice and glutamic-pyruvic transaminase, respectively. BUN, blood urea nitrogen. Scr, serum creatinine. iPTH, intact parathyroid hormone.

**Table 3:** Parameters of interest in the infection and non-infection groups.

	With COVID-19	Without COVID-19	*P-*value
ACE2 (ng/mL)^†^	1.08 ± 0.70	0.63 ± 0.13	**0.001**
ACE (ng/mL)^†^	914.5 ± 406.4	515.0 ± 354.6	0.258
ACE2/ACE^†^	2.28 ± 18.8	0.27 ± 0.77	**0.038**
TMPRSS2 (pg/mL)^†^	6.23 ± 18.56	17.2 ± 31.7	**0.042**
ACE2*TMPRSS2^†^	4.16 ± 2.90	0.96 ± 2.28	0.338

Results are given as mean ± SD. Differences between the groups were examined by *χ*^2^ test or unpaired *t-*test. *P* < 0.05: significant inter-group difference. ^†^Log-transformed values used in the analysis. Please refer to the text for the individual acronym.

Independent effect of the sACE2 and sTMPRSS2 level on the susceptibility was given in Table 4, as tested by the multiple logistic regression analysis. Clearly, effect of the sACE2 remained significant and that of sTMPRSS2 disappeared. In addition, contributory factors were lymphocyte count, the use of ARB, smoking, concurrent infection of HBV/HCV, dialysis session time, interdialysis weight gain and dialysis duration.

**Table 4:** Results of the likelihood ratio tests on the COVID-19 susceptibility.

	Model fitting criteria	Likelihood ratio tests		
Effect	−2 log likelihood of reduced model	Chi-square	df	Sig.
Intercept	100.510	0	0	
**Lymphocyte**	104.297	3.787	1	**0.052**
LogCRP	102.953	2.443	1	0.118
Log(uremic acid)	102.202	1.691	1	0.193
Log ferritin	102.105	1.595	1	0.207
**LogACE2**	107.221	6.711	1	**0.010**
LogTMPRSS2	101.923	2.731	1	0.102
Log(ultrafiltration)	103.510	1.917	1	0.821
**Use of ARB**	117.189	5.887	1	**0.041**
**Smoking**	107.002	6.492	1	**0.011**
**HBV/HCV**	108.527	8.017	2	**0.018**
**Each session time**	126.218	25.708	1	**0.000**
**Interdialysis weight gain**	125.562	7.119	1	**0.031**
**Dialysis vintage**	114.989	14.479	1	**0.000**

The chi-square statistic is the difference in -2 log-likelihoods between the final model and a reduced model. The reduced model is formed by omitting an effect from the final model. The null hypothesis is that all parameters of that effect are 0. LogCRP, log-transformed value used in the SPSS test. Please refer to the text for the individual acronym.

**Figure 1:**
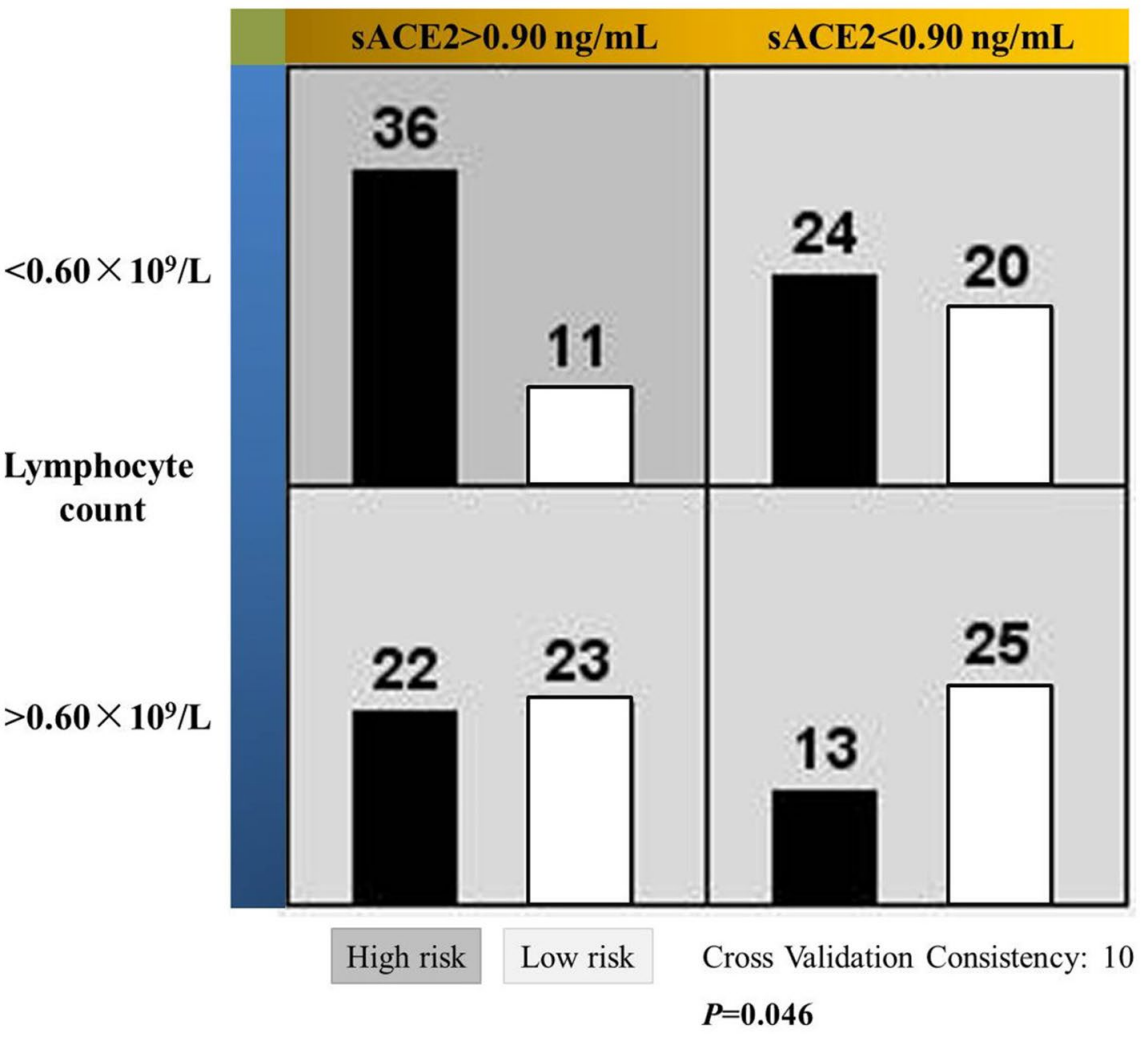
Significant interactive actions of the soluble ACE2 level and lymphocyte count on the susceptibility to COVID-19 in MHD patients. Left and right bars in each cell represent patients with and without the viral infection, respectively. Dark cell indicates higher risk of infection and the light ones for lower risk.

Next, we examined the usefulness of sACE2 and lymphocyte count in predicting susceptibility to COVID-19. For this purpose, these two indexes were processed by the ROC curve (Supplementary Figure S1). Accordingly, area under curve was 0.753 for sACE2 with a significance of 0.001 and the corresponding values for lymphocyte count were 0.730 and 0.003. Subsequently, cutoff values for sACE2 and lymphocyte count that yielded the most optimal paired sensitivity/specificity ranking were deduced as 0.90 ng/mL and 0.60 × 10^9^/L, respectively, using the Youden’s index. Eventually, the MDR method confirmed that patients with sACE2 level higher than 0.90 ng/mL and lymphocyte count lower than 0.60 × 10^9^/L were exposed to increased risk of infection (Figure 1).

## Discussion

In this study, we determined the plasma levels of sACE2 and sTMPRSS2 in the MHD patients, and confirmed the role of sACE2 in susceptibility to the COVID-19 infection. Next, a *de novo* predicting algorithm was configured using the sACE2 level and lymphocyte count. Then, contributory role of the use of ARB, smoking, concurrent HBV/HCV infection and single dialysis session time was defined. Arguably, an important link between the susceptible individual and COVID-19 was established, which can be useful in the cross-disciplinary prophylaxis of this zoonotic disease and the like.

The observation period was carefully selected for 2 weeks, considering the highly contagious nature of the COVID-19 and closely congregated setting of the in-center hemodialysis. Not surprisingly, the infected had a higher sACE2 level comparing with that of the non-infected, which was consistent with report confirming the role of sACE2 in promoting cell entry of the virus (20). This difference further led to higher sACE2/sACE ratio in the infection group and its association with disease severity was found in a recent study (21). Nonetheless, this ratio was not tested in the regression analysis because of possible co-linearity.

Less expected, sTMPRSS2 was significantly lower in the infection group, but the association was lost in multivariate analysis. In this regard, sACE2 was increased and sTMPRSS2 decreased in patients with eosinophilic asthma (22), whereas the same pattern of change in tissue expression was also observed in rats with decompensated chronic heart failure (23). We therefore speculated that soluble level and tissue expression of sACE2/sTMPRSS2 may be inversely related in some disease settings, as they are co-expressed ubiquitously in human cells (24). Supporting this speculation, we have previously found that NADPH oxidase, which was distributed in perivascular sympathetic nerve fibers, was functionally down-regulated during β2-adrenoceptor over-activity to help maintain renal function (25). Nonetheless, disassociation of the sTMPRSS2 with predilection to COVID-19 in our study may reflect the fact that ACE2 is also cleaved by other proteolytic enzymes such as a disintegrin and metalloproteinase-17 (ADAM-17) (26). Indeed, sACE2 but not sTMPRSS2 may predict disease severity (27).

Lymphopenia and reduced peripheral T cell levels was another major feature of the COVID-19, otherwise a normal immune response was supposed to be capable of resolving the infection (9). Compromised immune function may potentiate the virus’ power of infection for a given ACE2 level and vice versa. Therefore, COVID-19 infection was believed to be the result of close interaction between the virus and the immune system of an individual (28). Precisely, the near-significant association between lymphocyte counts and susceptibility (*p* = 0.052) in our work suggested that lymphocyte counts are *de facto* clinically relevant. Following these lines of evidence, we made the risk stratification by simultaneously using the sACE2 level and lymphocyte counts, which in turn produce a higher dimension of prediction than using either value alone. In this way, the risk of infection is considered as a binary function of cell entry and immune status. Our work thus offered new insights to uncover, in a broader sense, the pathogen-host interaction in the MHD patients.

It is noteworthy that the MDR method was used for this investigative purpose. It is basically a non-parametric method that facilitates the simultaneous detection and characterization of multiple genetic loci associated with a discrete clinical end-point. In our current work, different data partitions were achieved by the cutoff values of sACE2 and lymphocyte count (Figure 1), then assigned as presumed ‘genotypes’ and eventually tested for possible high-order interaction between these two variables. Using relatively small sample sizes, this method made it possible for data analysis in situations where traditional methods cannot be applied as we have previously described (25). In fact, performance of the MDR method was outstanding for skewed distributions over several current methods (29), including the principle component analysis which is the basic algorithm of dimensionality reduction in SPSS (30). Briefly, the MDR method is a useful multivariate non-parametric approach that can be used regardless of the factor distribution, the correlations between factors, and sample size. The related technical details are beyond the scope of our work and could be found elsewhere (29).

The risk factors also included the use of ARB, smoking, concurrent infection of HBV/HCV, each individual dialysis session time, interdialysis weight gain and duration on hemodialysis. In line with our findings, ARB was associated with a lower incidence of COVID-19 infection in both European MHD patients (10) and a mega-large population-based study (31). Possible explanations were attributed to the amelioration of inflammation and direct protection of the lung from the SARS-CoV-2 infection, despite the up-regulation of pulmonary ACE2 expression by ARB (32). As for the smoking, we had a somehow a counterintuitive finding. However, it was in agreement with data from United Kingdom, United States and France (31). The investigators believed that this may reflect a general immunomodulatory effect of smoking (31) or there may be a direct protective effect of nicotinic receptor stimulation (33).

Simultaneous presence of two pathogens is known to modulate, exacerbate or ameliorate, the effects of either or both. Reportedly, underlying HBV alone and HBV/HCV may decrease susceptibility to SARS-CoV-2 infection in a Korean nationwide survey and one Spanish study, respectively (34, 35). The use of antiviral agents including tenofovir was presumably accounted for these findings. Interestingly, HCV shared major similarities with the SARS-CoV-2, including utilization of the homoeomorphic ion channels (36). It is thus likely that the viruses may competitively seek utilization of the ion channels, which are diminished in the uremic state (37). The remaining three risk factors are all dialysis-specific and reduced individual hemodialysis session time may underlie the increased interdialysis weight gain. The latter in MHD patients is known to result in fluid overload or even edema of the lungs, both of which confer higher risk of pulmonary infection in ANCA-associated vasculitis (38) and acute brain injury (39). Evidently, patients undergoing long-term dialysis are at increased risk of the COVID-19 infection (40).

Age and gender has no effect on the circulating levels of sACE2/sACE/sTMPRSS2 in our MHD cohorts as a whole or divided into infection group and otherwise. Further, they were unrelated to the susceptibility to COVID-19. Reportedly, sACE was comparable between boys and girls up to 12-year and showed a boy predominance from age 15 (41). In an Arabian cohort, sACE2 was higher in male healthy individuals compared to female controls and this was reversed in those with type 2 diabetes (42). And there was a weak negative association with age but not gender in Japanese MHD patients (43). As the susceptibility to COVID-19 is concerned, age (>70) but not gender was a risk factors in the study including 38,236 MHD patients (10), whereas absence of age as risk factor was found in the French national dialysis registry (44) and a Chinese study observed a higher infection risk for patients aged 65-year or above (45). Taken together, the effects of these two demographic figures appeared to be maturation-dependent, epigenetics-specific (43) and hemodialysis-regulatory (46).

Our work is an essentially nested case–control study with strengths and inherent limitations. On the positive side, it was time-sensitive and marker-innovative, especially with definition of plasma levels of sACE2 and sTMPRSS2 in the MHD patients. Most recently, it was observed that ACE2-mediated pathway played an important role in the increased cardiovascular events during the ‘long COVID’ era (47) and a better understanding of such a role among MHD patients in particular may greatly ameliorate their risk of cardiovascular disease (48). Therefore, our work may definitely both warrant and facilitate further study on this critical issue in the MHD population. Admittedly, it by essence was a single-center study conducted in Chinese MHD patients, which requires caution when applied to other ethnic groups or general population. Further, there was non-availability of age-matched healthy controls in our study. By reality, recruitment of healthy controls was still impossible when the current work was initiated prior to the lifting of stringent quarantine policy, whereas age-matched ‘healthy controls’ without history of infection and vaccination of SARS-COV-2 after the open-up were scarce. Another setback was no free access to research facility having HPLC-MS instrument then. Likewise, enzyme inactive sACE2 was not considered in methodology. By practice, nonetheless, we did confirm that sACE2 could mediate cell entry of the SARS-CoV-2 (20) and its level may reflect membrane-bound mACE2 content (41), in addition to all the 16 publications validating the reliability of the Abcam Human SimpleStep ELISA^®^ Kit studying the pathogenesis of ACE2 in COVID-19 (49). Taken together, these limitations in methodology appeared to have no discernible effect on the outcome of the study and, in any case, close attention will be paid to them in our future work.

## Conclusion

Our model was able to pre-emptively predict the group of MHD patients with higher risk of SARS-COV-2 infection. Obviously, what counts is the methodology of configuring clinical parameters for prediction of viral diseases rather than the parameters *per se*. As such, this algorithmic approach may contribute to infection control in dialysis facilities after further validation in larger cohorts or randomized controlled trials. With no less certainty, such a screening model is needed as COVID-19 is not the first pandemic of this kind, nor is it the last.

## Data Availability

The original contributions presented in the study are included in the article/Supplementary material, further inquiries can be directed to the corresponding author.
