# Supplementary material for: Predicting susceptibility to COVID-19 infection in patients on maintenance hemodialysis by cross-coupling soluble ACE2 concentration with lymphocyte count: an algorithmic approach
Source: Front Med (Lausanne). 2024 Oct 30;11:1444719. doi: 10.3389/fmed.2024.1444719 (PMC11558530; doi:10.3389/fmed.2024.1444719)

**Supplementary Figure 1.**

Receiver operating characteristic curves of sACE2 and lymphocyte in the susceptibility of MHD patients to COVID-19 infection.


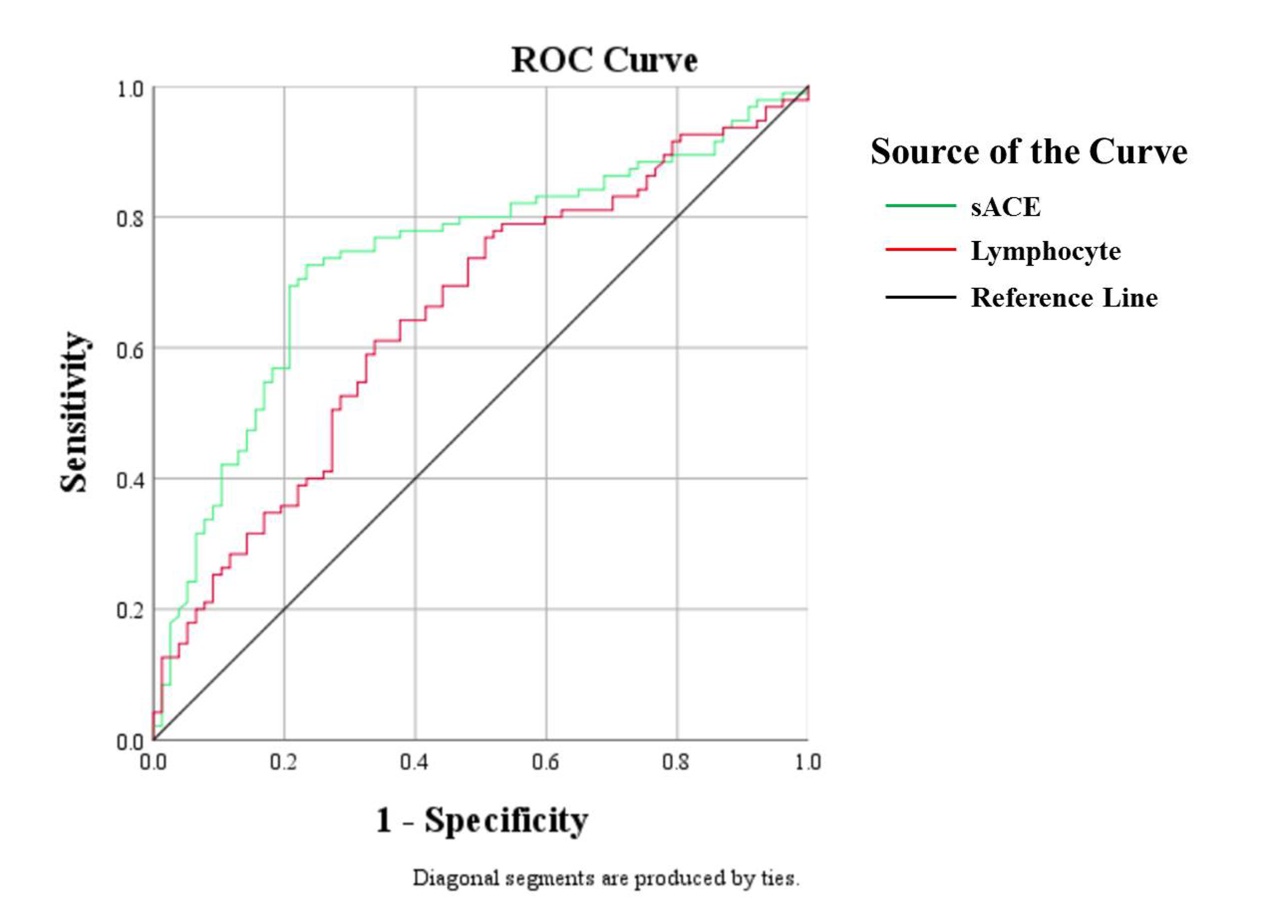

Supplement: Supplementary file 1 [file Data_Sheet_1.docx]
